# A Systematic Review of Technical Parameters for MR of the Small Bowel in non-IBD Conditions over the Last Ten Years

**DOI:** 10.1038/s41598-019-50501-9

**Published:** 2019-10-01

**Authors:** Jingyu Lu, Ziling Zhou, John N. Morelli, Hao Yu, Yan Luo, Xuemei Hu, Zhen Li, Daoyu Hu, Yaqi Shen

**Affiliations:** 1 Departments of Radiology, Tongji Hospital, Tongji Medical College, Huazhong University of Science and Technology, Wuhan, Hubei China; 2 Department of Radiology, The First Affiliated Hospital of Soochow University, Suzhou, Jiangsu China; 3 St. John’s Medical Center, Tulsa, OK United States

**Keywords:** Intestinal diseases, Magnetic resonance imaging

## Abstract

Technical guidelines for magnetic resonance imaging (MRI) of the small bowel (SB) in the setting of inflammatory bowel diseases (IBDs) were detailed in a 2017 consensus issued by European Society of Gastrointestinal and Abdominal Radiology (ESGAR) and European Society of Pediatric Radiology (ESPR); however, MRI for non-IBD conditions was not addressed. Hence, we performed a systematic review collecting researches on SB MRI for non-IBDs. The literatures were then divided into morphologic group and functional group. Information about the MRI techniques, gastrointestinal preparation, and details of cine-MRI protocols was extracted. We found that a 1.5 T MRI system, prone positioning, and MR enterography were frequently utilized in clinical practice. Gadolinium contrast sequences were routinely implemented, while diffusion-weighted imaging (DWI) was much less performed. The gastrointestinal preparation varied throughout the studies. No uniform protocols for cine imaging could be established. SB MRI examinations for non-IBDs are far from standardized, especially for functional studies. Recommendations for standard parameters in cine-MRI sequences are difficult to make due to lack of evidentiary support. MRI investigations in non-IBD conditions are needed and the standardization of non-IBD imaging in clinical practice is required.

## Introduction

Magnetic resonance enterography (MRE) or enteroclysis is an ideal technique to image the small bowel^1^. Cine-MRI is a helpful supplement to MRE as it provides a noninvasive way to access the global motility of small bowel (SB)^2^. Diffusion-weighted imaging (DWI) provides a quantitative functional evaluation of the SB without intravenous contrast, thus facilitating its use even in patients with impaired renal function^3^. MRE and MR enteroclysis have long been used to diagnose inflammatory bowel diseases (IBDs), especially Crohn’s disease (CD). The lack of ionizing radiation makes it preferable to CT for this purpose, particularly in patients under 35 who often require multiple scans to assess disease progression^4^. DWI^5–7^ and contrast-enhanced T1W sequences^8–10^ have been widely utilized to monitor the activity and remission of CD. In distinction, the role of MRE and MR enteroclysis in non–IBD conditions such as neoplasm, SB obstruction, diverticular disease, and functional disorders is far less described^11^.

With the transition toward quantitative imaging and greater demand for multi-center cooperation, medical image standardization has become increasingly important. In 2016, The European Society of Gastrointestinal and Abdominal Radiology (ESGAR) and European Society of Pediatric Radiology (ESPR) released a consensus statement on the technical performance of cross-sectional imaging of IBDs^12^. Recommendations concerning patient preparation and image acquisition protocols for MRE and MR enteroclysis in the imaging of IBD were made based on the available literature. Although the relationship between MRI image features and pathological features has been studied thoroughly^7,13–15^, further strong evidence on protocol optimization is still needed. The consensus statement highlighted that various protocols and sequences have been utilized to study IBDs with MRE and MR enteroclysis. In this paper, we review original studies on MRE and enteroclysis of non-IBD small bowel disorders in last ten years, including studies performed on healthy volunteers, with the intent of providing a reference for future standardization.

## Methods

### Literature search

A systematic search for studies focusing on small bowel MRI exclusive of IBDs published from January 2008 to December 2018 was performed. Published articles in English language journals were identified in electronic databases by using MeSH. The search terms were as follows: ‘small intestinal and magnetic resonance imaging not (Crohn disease and inflammatory bowel disease)’. Only original research studies written in English in the past 10 years were included. The search resulted in a total of 374 articles in PubMed Central, 344 in Web of Science and 352 in Medline.

### Inclusion criteria and selection process

**Figure 1:**
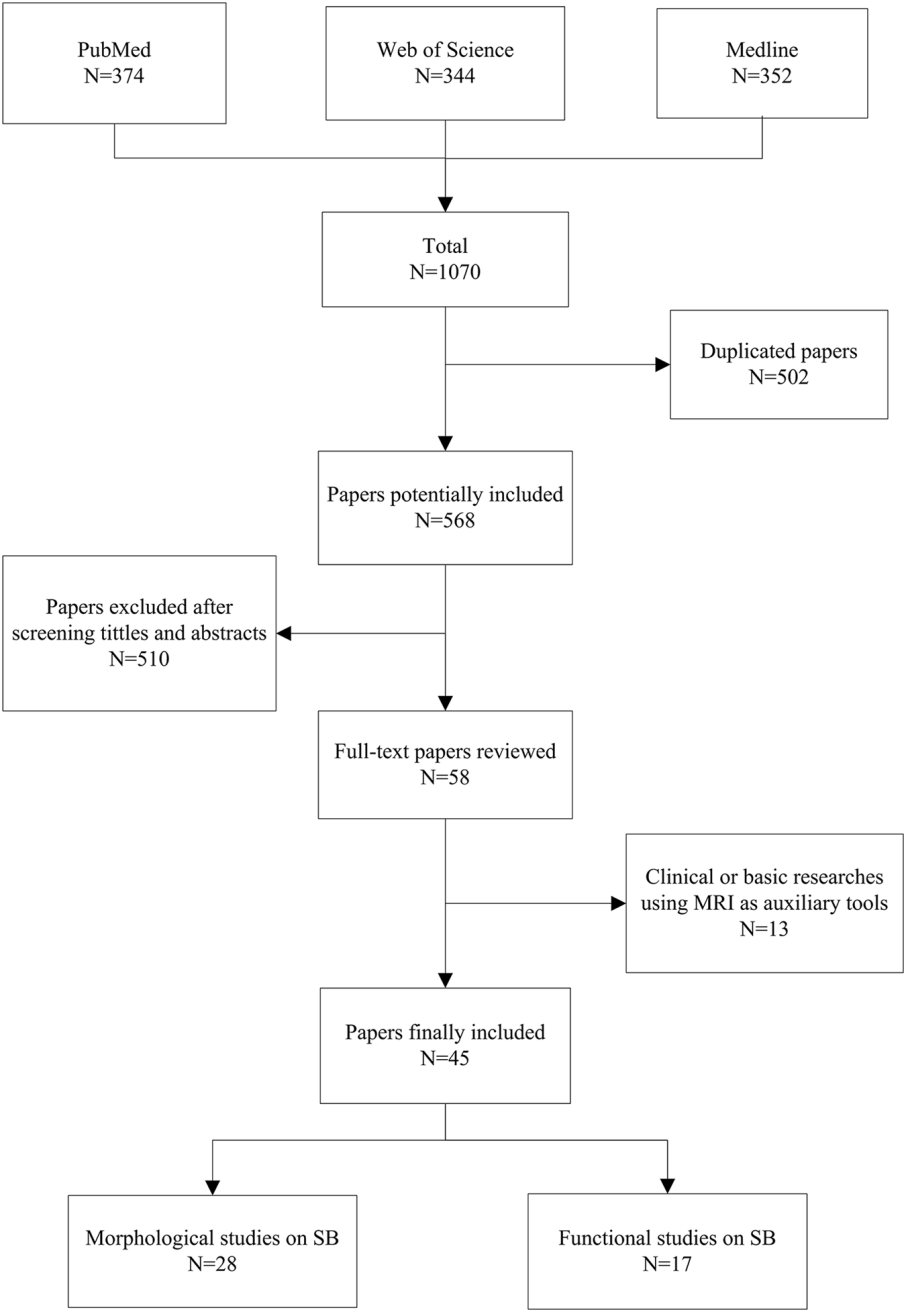
Flowchart of the process of selecting articles.

After removing duplicated papers, the remaining 568 papers were initially reviewed by two participants based on article titles and available abstracts. The exclusion criteria were as follows: research focused on Crohn disease, research focused on other IBDs, animal research, and case reports. After the exclusion of 510 papers, the full text of 58 articles was evaluated. 13 additional articles were then excluded as they were found to be clinical or basic science research using MRI as an ancillary tool. In total, 45 articles were included (Fig. 1).

### Data extraction

The selected studies were divided into two groups according to the purpose of the research. The morphological group included studies investigating organic small bowel abnormalities and the functional group consisted of those focused on intestinal function. All studies were sorted according to author and year, study population, examination procedures, MR protocols, and other relevant factors.

## Results

### Examination procedures

**Figure 2:**
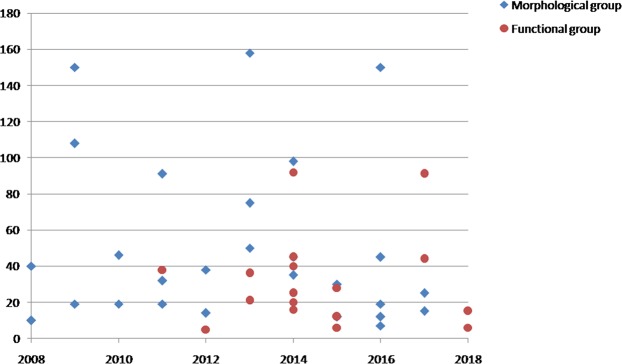
Graph shows the sample size of each studies included (morphological and functional group) since 2008.

The morphological group consisted of 28 articles and the functional group of 17 articles. The sample size examined in both articles was approximately around 30 for both groups (Fig. 2). In the morphological group, the pre-scan fasting time ranged from 4 hours to 8 hours; the functional group was similar, ranging from 4 hours to 9 hours. The interval between small bowel distension and MR acquisition was 0.5 to 1 hour in both groups (Table 1).

**Table 1:** General information of the small bowel MRI technique in studies included.

		Morphological group	Functional group
Fasting		4–8 h	4–9 h
Volum of enteral contrast agents	MR enterography	900–2000 ml	1000–1350 ml
	MR enteroclysis	1000–3000 ml	/
Interval time		0.5–1 h	0.5–1 h

Interval time, Interval time between small bowel distension and MR acquisition.

### MR system and protocol

In the morphological group, 22 studies were conducted using 1.5 T MR scanner, one was conducted using a combination of 1.5 T and 3.0 T and two making a comparison between 1.5 T and 7.0 T. In the functional group, 13 studies were conducted at 1.5 T and 4 studies at 3.0 T. In the group of morphological studies, 23 of the 28 studies adopted contrast-enhanced sequences. Only 5 of the 28 utilized DWI. Ten of the fifteen studies in functional group utilized cine-MRI to assess the small bowel function (Table 2).

**Table 2:** Summary information of the small bowel MRI technique in studies included.

		Morphological group (N)	Functional group(N)
Total		28	17
MR system	1.5 T	22	13
	3.0 T	0	4
	1.5 T & 3.0 T	1	0
	1.5 T & 7.0 T	2	0
	NA	3	0
Technique	MRE	18	13
	MR Enteroclysis	6	0
	MRE & MR Enteroclysis	3	0
	NA	1	4
Position	Prone	12	5
	Supine	4	5
	Prone & Supine	2	0
	NA	10	7
Gd based contrast agents		23	/
DWI		5	/
Cine MRI		1	11

N, Number of articles utilizing the techniques listed in the table; NA. Not Available.

### Enteric contrast agents

In the morphological group, 96% of the studies distended the small SB before MRI acquisition with enteric contrast. In the functional group, 71% of studies mentioned the use of enteric contrast agents. The most frequently used agents were polyethylene glycol (PEG, n = 12), mannitol hydrosolution (n = 4), and methylcellulose (n = 4) in the morphological group. In functional group, mannitol hydrosolution (n = 7) was the most commonly used enteric contrast agent (Tables 1 and 3).

**Table 3:** Administration of enteric contrast agents and antiperistaltic agents in studies included.

			Morphological group(N)	Functional group (N)
Total			28	17
Enteric contrast agents	Polyethylene glycol (PEG)		12	0
	Mannitol hydrosolution		4	7
	Methylcellulose		4	0
	Locust bean gum &mannitol hydrosolution		3	0
	Sorbitol or psyllium		1	0
	Dilute barium sulfate		1	1
	Green tea		1	0
	Water		0	3
	NA		2	6
Antiperistaltic agents*	Hyoscine butylbromide or anisodamine	10–20 mg	16	/
		20–60 mg	4	/
	Glucagon	0.5–1 mg	5	/
	Tiemonium methylsulphate	0.5 mg	1	/
	NA		4	/

N, Number of articles administrating the agents listed in the table; NA, Not Available; *One article could use multiple antiperistaltic agents; “/” on behalf of not doing statistics.

### MR enterography and enteroclysis

In the morphological group, MR enterography was applied in 21 studies while MR enteroclysis was applied in 9 studies. MR enterography was carried out in 13 functional studies. No studies inserted a nasoduodenal catheter under fluoroscopy in the functional group (Table 2). In the morphological group, 900 ml to 2000 ml contrast material was orally administered in the studies carrying out MR enterography while 1000 ml to 3000 ml of enteric contrast agents was administered for MR enteroclysis. In the functional group, 1000 ml to 1350 ml oral administration was used to achieve small bowel distension (Table 1).

A study by Lawrance *et al*.^16^ showed equivalent distal small bowel distension and artifacts between MR enteroclysis and MR enterography. In terms of the proximal small bowel, MR enteroclysis achieved a greater degree of distension; however, the diagnostic performance of the two techniques was equal. A previous paper suggested 900 ml of oral contrast agent was sufficient to obtain duodenal distension, and 1350 ml was sufficient to distend the distal jejunum and ileum^17^.

### Position

Prone position was adopted in 12 studies and supine position in 4 studies for the morphological group. In the functional group, 5 studies explicitly mentioned the use of a prone position, while supine position was mentioned in 5 studies (Table 2). In an MR enterography investigation by Cronin *et al*.^18^ comparing the two positions, superior small bowel distention was achieved with prone positioning, but this did not result in statistically significant differences in lesion detection. However, in the surveillance of patients with Peutz-Jeghers syndrome (PJS), Maccioni *et al*.^19^ found that a combination of supine and prone position was significantly more accurate than supine position alone for the detection of polyps smaller than 15 mm. In cine-MRI studies, prone positioning could limit anteroposterior displacement of SB loops out of the coronal slice and make quantitative motility analyses more reliable^20^.

### Antiperistaltic agents

Four types of antiperistalic agents were mentioned in morphological group. 10–20 mg hyoscine butylbromide or anisodamine (654–2) was prescribed in 16 studies; 4 studies utilized greater than 20 mg hyoscine butylbromide (20–60 mg). Five studies administered 0.5–1.0 mg glucagon to inhibit the peristalsis of small bowel (Table 3). In one study, 0.5 mg tiemonium methylsulphate was administered in place of glucagon in nine patients. According to a review by G. Masselli *et al*.^1^, antiperistaltic agents are optimally administered intravenously just before the start of MR examination, and in patients who receive intravenous contrast, a second dose of antiperistaltic agents at the same strength should be given before contrast injection. The role of a sublingual antiperistaltic agent, hyoscyamine sulfate was investigated in one cine-MRI study but the extent of its impact on the evaluation of cine MR enterography is still uncertain^21^.

Though the additional value of spasmolytic administration to improve image quality of diffusion-weighted sequences has not been evaluated, Elisa *et al*.^22^ hypothesized that image quality can be increased by administrating a spasmolytic agent before the DWI MR sequence. However, in one study by Taro^23^, mean apparent diffusion coefficients (ADCs) before butylscopolamine administration were statistically significantly different from those after butylscopolamine administration.

As antiperistaltic agents were not routinely used for the evaluation of small bowel function, their use was not evaluated in the functional group.

### The application of DWI

In the studies by Schmidt^24^ and Amzallag-Bellenger^22^, b values of 0 and 800 s/mm^2^ were used for DWI, while in Low *et al*.^25^ a b-value of 500 s/mm^2^ was utilized. In Plumb *et al*.^26^, DWI sequences were obtained with b-values of 0, 50, 150, 300, 600 s/mm^2^. In a study by Takahara^23^, DWI with b-values of 0 and 50 s/mm^2^ was evaluated, and low b-value DWI found useful in distinguishing strangulated from non-strangulated intestinal obstructions. The use of high b values (b >800–1000 sec/mm^2^) can help negate the high signal intensity of bowel contents and normal bowel mucosa^27^. Amzallag-Bellenger *et al*.^22^ reported significantly increased detection of small bowel tumors by junior radiologists after adding DWI sequences to traditional MRI. All studies included with DWI were performed at 1.5 T.

The application of DWI to intestinal neoplasms could provide useful information about TNM stage and treatment response^27^. In a comparative study by Wong^28^, DWI was found to be comparable to PET/CT in diagnosis and treatment response evaluation in patients with gastrointestinal stromal tumors (GISTs). Tang *et al*.^29^ found the non-Gaussian fractional order calculus diffusion model may be able to predict early GISTs response to second-line sunitinib targeted therapy.

### Cine – MRI

The cine-MRI protocols for 13 studies are shown in Table 4. Breath-hold scanning and imaging in the coronal plane are most commonly adopted in cine-MRI studies. The parameters of slice, field of view (FOV), matrix size, and temporal resolution varied widely. Post-processing was also variably performed from manual measurements to semi-automated evaluations utilizing computer software.

**Table 4:** Parameters of Cine-MRI studies included.

Author, Year	Breath	Oriention	Number of slices	Sclice thickness (mm)	FOV (cm)	Matrix	Temporal resolution	Scan time
de Jonge 2018	BH	Cor	1	10	40	160	10 frames/s	20 s
Khalaf 2018	FB	Cor	1	10			1 s	60 s
Fuyuki 2017	BH	Cor	3	10	38	256	0.5 s	16 s
Bickelhaupt 2015	BH	Cor	multiple	3	14.4	68		60 s
Bickelhaupt 2015	BH	Cor, Sag	multiple	10	40	256	550 s	40 s
Bickelhaupt 2014	BH	Cor	15–25	10	40	256	550 s	17 s/slice
Ghobrial 2014		Cor		8	36–40	160–268	1–2 s	
Menys 2014	BH FB	Cor	6 slices in a volum	30/volum	42		1 volume/s	20 s/slice 1 min FB
Bickelhaupt 2014	FB	Cor	multiple	10	48	256	250 ms	
Menys 2013	BH	Sag, Cor, Axi	multiple		42		1 volume/s	
Ohkubo 2013	BH	Cor	3	10	38	256	0.5 s	16 s
Takahara 2011	BH	Cor	1	10	38	192	0.9 s	20 s
Takahara 2011	BH	Cor	1	10	38	192	0.98 s	20 s

FOV, Field of view; BH, Breath-hold; FB, Free-breathing; Cor, Coronal; Sag, Sagittal; Axi, Axial.

Breath-hold scanning can reduce respiratory motion artifacts but limits the duration of the motility sequences. Free-breathing techniques could prolong acquisition times of the cine sequences which may prove beneficial as long duration cine-MRI is more reliable^30^. To observe consistent small bowel motility, a temporal resolution of at least 1 frame per second and a duration of at least 15 s is necessary in breath-hold scans^31^. The coronal plane is routinely used to analyze small-bowel motility as it enables adequate coverage of the entire small bowel and is optimal for assessment of peristalsis^30,32,33^. Regardless of the placement of coronal slices within the ventral, central, or dorsal portion of a small bowel segment, parameters reflecting SB movement did not change^33^. In a study of small bowel motility in 21 healthy volunteers, software-quantified measurements were repeatable and sensitive to changes in SB activity induced by pharmacologic manipulation^34^. However, in a study of 20 healthy volunteers, large variations in segmental motility were found within the same individual at the same time and the replicability was poor at the same location over time^35^. Studies by Bickelhaupt *et al*. in which free-breathing MRI was adopted showed that software-assisted evaluation of small bowel motility was much faster, more accurate, and reproducible than manual assessment^36^. Two mathematically established methods, Lomb-Scargle and Sinus-Fitting were reliable in the automated assessment of small bowel contraction frequency^32^. One optimized technique based on registration methods showed excellent agreement between observers for the analysis of overall gut motility in unprepared small bowel conditions^37^.

### Clinical indications

#### Small-bowel neoplasms

Patients with PJS may suffer from polyp-related symptoms caused by gastrointestinal intussusception, obstruction, or infarction in addition to an increased risk of cancer^38^. MRE or MR enteroclysis could be used for periodic small bowel surveillance^39^. MR enteroclysis was found to have comparable diagnostic performance to double balloon enteroscopy (DBE) for polyps >15 mm, but DBE was better tolerated than MR enteroclysis by most patients^40^. A comparison between MR enterography and capsule endoscopy showed that although MR enterography was less comfortable for the patients, it was more reliable in measuring polyp size and less likely to miss large polyps^41^.

Neuroendocrine tumors (NETs) are common small bowel malignancies, the incidence of which is increasing^42–44^. Small bowel NETs are highly vascular tumors^45^. MR-enterography achieved a sensitivity of 95% for the detection of small bowel NETs. Contrast-enhanced 3D VIBE sequences were more sensitive than HASTE and True FISP for detection of NETs^46^.

GISTs are the most common mesenchymal tumors of the gastrointestinal tract^47^. MRI features and ADC measurements can be used to predict the NIH risk stratification. Obvious enhancement with intratumoral cystic change and lower ADC values may indicate a higher risk grade tumor^48–50^. GISTs could be effectively controlled by imatinib and need regular imaging follow-up. Tang *et al*.^51^ found lower baseline ADC and marked ADC increase at one week after therapy were associated with good response to imatinib mesylate.

In patients with peritoneal dissemination from appendiceal malignancy after surgical cytoreduction (CRS) and hyperthermic intraperitoneal chemotherapy (HIPEC), MRI detects tumor recurrence earlier than serum tumor makers alone and with greater accuracy^25^.

Amzallag-Bellenger *et al*.^52^ found that intravenous administration of gadolinium-based contrast improved sensitivity of SB tumor detection especially in patients with poor SB distension. Meanwhile, they found MR enterography achieved high negative predictive values (NPV) up to 98% on a perpatient basis, a factor conductive to excluding SB tumor when findings at capsular endoscopy are equivocal. In one MR enteroclysis study, overall sensitivity, specificity, and accuracy in identifying patients with small-bowel neoplasms were 91%, 95% and 95%, respectively^53^. The overall concordance between MR enteroclysis and histological examination of the surgical specimens was 62%^54^.

#### Small bowel obstruction

Computed tomography (CT) is the first-line examination for patients suspected of small bowel obstruction (SBO), but the diagnosis of strangulation or ischemia remains difficult^55–58^. Low b-value MRI obtainable in a short acquisition time (less than 60 s) might provide a feasible modality to detect bowel loops with compromised blood supply: the mean ADCs of the closed loop, near the site of obstruction, and distant from the obstruction site are all significantly different from each other^23^. Takahara *et al*.^59^ described the “peristalsis gap sign” referring to an akinetic or severely hypokinetic closed loop on cine MRI. This finding achieved higher sensitivity, specificity, positive predictive values (PPV), and NPV for strangulated small bowel than CT.

In studies of chronic intestinal pseudo-obstruction (CIPO), the diagnostic value of mean luminal diameter (MLD), contraction ratio (CR), and contraction cycle (CC) extracted from cine-MRI were evaluated. Results showed that MLD and CR differed significantly between patients with CIPO and healthy volunteers^60,61^. CC was significantly slower in CIPO patients with impaired small intestinal peristalsis compared to those without^60^.

#### Obscure gastrointestinal bleeding

Obscure gastrointestinal bleeding (OGIB) is defined as persistent or recurrent bleeding of unknown origin after negative endoscopy^62^. One comparison study between MR enteroclysis and capsule endoscopy in adults found that although MRE had a low accuracy in diagnosing OGIB caused by angiodysplasia and duodenal ulcers, its diagnostic performance in detection of OGIB caused by CD, SB tumor, and Meckel’s diverticulum was satisfactory^63^. Casciani *et al*.^64^ found MRE to be a safe imaging modality for the evaluation of pediatric OGIB with diagnostic performance comparable to capsule endoscopy in a group of 25 pediatric patients.

#### Irritable bowel syndrome (IBS)

Lam *et al*.^65^ assessed small bowel and colonic regional volumes as well as gut transit with MRI and found fasting small bowel water content in IBS patients with constipation to be significantly less than those of healthy volunteers. Fasting transverse colon volumes in IBS patients with constipation were significantly greater and whole-gut transit times were prolonged compared with patients without constipation and healthy volunteers.

The clinical presentation of small intestine bacterial overgrowth (SIBO) often overlaps with that of IBS. SIBO is characterized by increased fermentation of carbohydrate substrate due to bacterial contamination of the small intestine^66,67^. MRI can assist in the differential diagnosis of IBS and SIBO by synchronously evaluating oral to caecal transit time (OCTT) and median small bowel gas volume (SBGV)^68^.

#### Short bowel syndrome (SBS)

SBS is characterized by the inability to maintain acceptable nutrition due to surgical resection, congenital defect, or disease-related loss of absorption^69^. Although it is difficult to noninvasively measure the physical length of small bowel, MRE provides us with a promising approach. Sinha *et al*.^70^ applied a vascular imaging software to estimate small bowel length on true FISP sequences and found a significant correlation with surgical measurements. Wilson *et al*.^71^ found a custom-designed algorithm to be feasible and accurate for calculation of small intestine length on true FISP images. Application of automatic software and machine learning to measure SB length may further improve these techniques, which could benefit patients with or at risk for SBS in nutritional management and surgical approach.

#### Other SB abnormalities

One study by Cobelli *et al*.^72^ reported that dynamic contrast-enhanced (DCE) MRI of the small bowel could be used to investigate mesenteric vascular flow finding impaired perfusion to be a reliable MRI marker in paroxysmal nocturnal hemoglobinuria (PNH) patients with abdominal pain.

MR enteroclysis and MR enterography have the ability to diagnose SB diverticulitis and diverticulosis; although, the superiority of MRI to other modalities in this regard is not clear. Focal inflammatory changes of the mesenteric fat, asymmetric wall thickening and the presence of multiple diverticula are keys to differentiate SB diverticulitis from other SB IBDs and neoplasms^73^.

Lymphoid nodular hyperplasia is an incidental finding and normal variant particularly in children and adolescents but can mimic CD on MR enterography. Image features including T2 signal, enhancement pattern, wall thickness, and ADC values showed no significant difference between lymphoid nodular hyperplasia of the terminal ileum and CD^26^.

## Conclusion

MRI techniques for SB imaging differ between different institutions especially with respect to gastrointestinal preparation and cine-MRI scanning. Polyethylene glycol (PEG) and mannitol hydrosolution were the most commonly utilized enteric contrast agents. Prone positioning was more commonly used than supine; although, the balance of patient comfort versus detection rate for pathology requires further study and literature support. DWI was not routinely used for MR assessment of non-IBDs, and its role in detecting non-IBD pathology especially SB neoplasms is not yet clear. MRI protocols for cine-MRI of the small bowel vary widely, and the definition of normal and abnormal SB motility by this modality is not clear. The consensus guidelines issued by ESGAR and ESPR can be used as a reference for non-IBD small bowel MRI; however, development of standardized protocols for cine-MRI sequences will require further evidence from original research.

## Supplementary information

Supplementary table
