# Supplementary material for: A Systematic Review of Technical Parameters for MR of the Small Bowel in non-IBD Conditions over the Last Ten Years
Source: Sci Rep. 2019 Oct 1;9:14100. doi: 10.1038/s41598-019-50501-9 (PMC6773732; doi:10.1038/s41598-019-50501-9)
Supplement: Supplementary file 1 — Supplementary table [file 41598_2019_50501_MOESM1_ESM.docx]

**A Systematic Review of Technical Parameters for MR of the Small Bowel in non-IBD Conditions over the Last Ten Years**

Jingyu Lu^1^ MD, Ziling Zhou^1^ MD, John N. Morelli^2^ MD, Hao Yu^1^ MD, Yan Luo^1^ MD, Xuemei Hu^1^ MD, Zhen Li^1^ MD, PhD, Daoyu Hu^1^ MD, PhD, Yaqi Shen^1^ MD, PhD

1 Departments of Radiology, Tongji Hospital, Tongji Medical College, Huazhong University of Science and Technology, Wuhan, Hubei, China.

2 St. John’s Medical Center, Tulsa, OK, United States

**Corresponding Author:**

Please address correspondence to Yaqi Shen M.D., Ph.D.

**Address**: Department of Radiology, Tongji Hospital, Tongji Medical College, Huazhong University of Science and Technology, 1095 Jiefang Avenue, Wuhan, Hubei, China. 430030

**E-mail**: [**yqshen@hust.edu.cn**](mailto:yqshen@hust.edu.cn)

**Supplementary Table 1** Overall information of the small bowel MRI technique in morphological studies included

| **Author,Year** | **Disease** | **Samplesize** | **Age** | **Scaner** | **Fasting** | **Enteric contrast agents** | **Interval time** | **Antiperistaltic agents** |
| --- | --- | --- | --- | --- | --- | --- | --- | --- |
| Goverde 2017▲ | PJS | 15 | 22-64 |  |  | 1000 to 3000mL methylcellulose (0.5%) water solution |  | Butylscopolaminebromide 20mg before the injection of gadolinium |
| Casciani 2017△○ | OGIB | 25 | 0.33-16 | 1.5 T  Siemens | 4 h | PEG water solution  10 mL/kg body weight | 90 min | 10 mg of hyoscine butylbromide |
| Dohan 2016△○ | NET | 19 | 48–87 | 1.5 T  Siemens | 6 h | 1.5 L of an iso-osmotic solution of water mixed with PEG and electrolytes | 45 min | 0.5 mg of glucagon or tiemonium methylsulphate 1 min before the start of 3D VIBE |
| Schmidt 2016▲△ | SBD | 45 | 12–71 | 1.5 T  Siemens |  | different oral contrast |  | 10mg of N-butylscopolamine |
| Hahnemann 2016△○ | HV | 12 | 21.5–31.8 | 1.5 T  Siemens 7 T  Siemens | 4 h | 1 L of a locust bean gum/mannitol hydrosolutio | 45 min |  |
| Masselli 2016△ | SBD | 150 | 18-74 | 1.5 T  Siemens |  | 1400ml PEG water solution | 45 min | 10 mg of scopolamine-N-butylbromide |
| Mansoori 2016▲△● | diverticulitis | 7 |  | 1.5 T  Siemens |  | 900 ml of a neutral biphasic enteric contrast agent orally or 1.5–2 L of 0.5 % methylcellulose solution administered via NJT | 45–60 min | 40–60 mg butylscopolamine or 1 mg of glucagon |
| Pei-You 2015△ | SBD | 30 | 24-67 |  |  | 1500-2000 ml isotonic mannitol | 30 min | 20mg anisodamine (654-2) |
| Hahnemann 2015△○ | HV | 12 | 21–31 | 1.5 T  Siemens 7 T  Siemens | 4 h | 1000 mL of a 0.2% locust bean gum and 2.5% mannitol hydrosolution | 45 min | 20 mg N-butylhyoscine twice |
| De Cobelli 2015△● | PNH | 12 | 21-59 | 1.5T  Philips | 6 h | 1.5 L of a PEG solution | 30 min | 20 mg of Scopolamine-butylbromide before perfusion data acquisition |
| Plumb 2014△○ | SBD | 35 | 13-21 | 1.5 T  Siemens |  | 1–1.5 L 2.5% mannitol | 40 min | Hyoscine butylbromide |
| Amzallag-Bellenger 2014△○ | SBD | 98 | 18-85 | 1.5 T  Siemens | 6 h | 1500 ml of an isoosmotic solution of water mixed with PEG and electrolytes | 45 min | 0.5 mg of glucagon before the start of dynamic T1-weighted sequences. |
| Saini, S. 2014△○ | HV | 45 | 18–85 | 1.5-T  Philips 3 T  Siemens | overnight | 1.5 l of 3% sorbitol or  2 l of 1.6 g/kg psyllium | 45 min 2 h | 40mg butyl scopolamine 20mg intramuscular and 20 mg IV. |
| Low 2013△● | appendiceal neoplasm | 50 | 47 | 1.5-T GE |  | 1200 ml of dilute barium sulfate | 45 min | Glucagon 1 mg IV or Levsin 0.25 mg IV |
| Amzallag-Bellenger E 2013△○ | SBD | 75 | 19–85 | 1.5 T  Siemens | 6 h | 1,500 ml of an iso-osmotic solution of water mixed with PEG and electrolytes | 45 min | 0.5 mg glucagon 1min before T1 |
| Pappalardo  2013▲△○ | SB neoplasms | 158 | 42-80 | 1.5-T GE |  | 1600–2000 mL of PEG water solution | 45 min | 10 mg hyoscine butylbromide |
| Wiarda1 2012▲ | OGIB | 38 | 28–75 |  |  | 1–3L 0.5% methylcellulose solution |  | 20mg butyl scopolamine before the injection of gadobutrol |
| Maccioni 2012△○● | PJS | 14 | 27-52 | 1.5 T  Siemens |  | 2 L of PEG | 30 min | 20 mg of Hyoscine Butylbromide twice  10 mg immediately before the examination and 10 mg before the injection |
| Silit E 2011▲ | SBD | 32 | 18-66 | 1.5 T  Siemens |  | 1500– 2000 ml of 0.5% methylcellulose solution |  | 20 mg hyoscine butylbromide |
| Takahara 2011 | HV SBO | 5 14 | 28–44 24–79 | 1.5T  Philips |  | 1000 mL of green tea |  | 1 mL 1% butylscopolamine |
| Weyenberg  2010▲○ | SBD | 91 | 18–83 | 1.5 T  Siemens |  |  |  |  |
| Bocker U 2010△ | SBD | 20 21 5 | 17–78 | 1.5 T  Siemens |  | 2 l of a 2.5% mannitol solution | 1h | 20–40 mg of N-butylscopalamine |
| Gupta 2010△ | PJS | 19 | 21-67 | 1.5 T  Siemens | restricted to clear fluids | 1.5 L 2.5% mannitol and 0.2% locust bean gum | 1h | 20-mg dose of hyoscine butylbromide after the localizing scans  before the contrast-enhanced sequences |
| Masselli 2009▲○ | SB Neoplasms | 150 | 17–84 | 1.5-T GE |  | 1600–2000 mL of PEG water solution and electrolytes |  | 20 mg of hyoscine butylbromide |
| Crooka 2009△○ | SBD | 19 | 25–83 | 1.5 T  Siemens | overnight | 1 l of a 3% mannitol solution | 1h | 20 mg of N-butyl-scopolamine twice |
| Lawrance 2009▲● | SBD | 108 |  | 1.5 T  Siemens | 6h | 1000 mL of PEG orally 800-2000 mL via a nasojejunal tube (NJT) |  | 10 mg intravenous hyoscine butylbromide |
| Lohan 2008△ | SB lymphoma | 10 | 44–85 | 1.5 T  Siemens | 8 h | a single sachet of l PEG diluted in 1000 mL of water | 45 min |  |
| Cronin 2008△○● | SBD | 40 | 15–73 | 1.5 T  Siemens | 8 h | 1000 ml PEG solution | 10-15 min  30 min |  |

PJS = Peutz-Jeghers Syndrome; OGIB = Obscure Gastrointestinal Bleeding; NET = Neuroendocrine tumors; SBD = Small Bowel Disease; HV = Healthy Volunteers; PNH = Paroxysmal Nocturnal Hemoglobinuria; IV = intravenous; PEG = polyethylene glyco

△= Enterography, ▲= Enteroclysis, ○=Prone, ● = Supine

**Supplementary Table 2** Overall information of the small bowel MRI technique in functional studies included

| **Author, Year** | **Disease** | **Sample Size** | **Age** | **Scaner** | **Fasting** | **Enteric contrast agents** | **Interval time** | **Antiperistaltic agents** |
| --- | --- | --- | --- | --- | --- | --- | --- | --- |
| De Jonge  2018△● | HV | 6 | 21-25 | 3.0 T Philips |  | 1000 ml of 2.5% mannitol | 10 min |  |
| Khalaf  2018△ | HV | 15 | 29 ± 10 | 1.5 T Philips |  |  |  |  |
| Lam 2017● | IBS | 91 | 21-65 | 1.5 T Philips |  |  |  |  |
| Fuyuki 2017△ | CIPO HV | 33 11 | 16-79 | 1.5 T Philips |  | 1000 ml of water orally |  |  |
| Savarino 2015● | HV | 28 | 30±8 | 1.5 T GE |  |  |  |  |
| Bickelhaupt 2015△○ | HV | 6 | 25–53 | 3.0 T Philips | 4 h | 1000 ml of 2.5% mannitol | 1 h |  |
| Bickelhaupt 2015△○ | HV | 12 | 25–55 | 1.5 T Philips | 4 h | 1000 ml of 3% mannitol | 1 h |  |
| Bharucha 2014△ | HV | 40 | 37 ± 3y 31±3y | 1.5 T GE | 6 h | 1350 ml low concentration barium solution | 45 min |  |
| Bickelhaupt 2014△○ | SBD | 45 | 22-87 | 1.5 T Siemens | 4 h | 1000 ml of 3% mannitol | 1 h |  |
| Ghobrial 2014△ | SBD | 92 |  | 1.5 T Philips  1.5 T Siemens | 4 h | 1350 ml of a stabilized suspension for bowel luminal contrast | 24 min | 0.5mg sublingual (SL) hyoscyamine sulfate. |
| Murray 2014● | HV | 16 | 24 ± 5 | 1.5 T Philips |  |  |  |  |
| Menys A 2014△ | HV | 20 | 22–48 | 3.0 T Philips | 4 h | 11 of 2% mannitol solution | 50 min |  |
| Bickelhaupt 2014△○ | SBD | 25 | 18–68 | 1.5 T GE |  | 1000 mL of 3% mannitol |  |  |
| Menys A 2013△○ | HV | 21 | 22–48 | 3.0 T Philips | 4 h | 1 L of a 2% mannitol solution | 50 min | 0.5 mg neostigmine OR 20 mg intravenous butylscopolamine |
| Ohkubo 2013△ | HV IBS CIPO | 12 12 12 | 31-78 24-87 25-87 | 1.5 T Philips |  | 1000 ml of water just before the MRI examination |  |  |
| Farghal1 2012△● | HV | 5 | 32-48 | 1.5 T Siemens | 9 h | 1 L of tap water | 30 min | 20 mg of Hyoscine Butylbromide intramuscularly |
| Takahara 2011 | SBO | 38 | 23–83 | 1.5 T Philips |  |  |  |  |

﹡IBS = Irritable Bowel Syndrome; CIPO = Chronic Intestinal Pseudo-obstruction; SBD = Small Bowel Disease; HV = Healthy Volunteers

△= Enterography, ▲= Enteroclysis, ○=Prone, ● = Supine
